# Incidence of Human Visceral Leishmaniasis and Social Vulnerability in an Endemic Area of Northeastern Brazil: A Time Series Analysis

**DOI:** 10.3390/epidemiologia7030066

**Published:** 2026-05-07

**Authors:** Karen Brayner Andrade Pimentel, Romário de Sousa Oliveira, Laércio Viana Oliveira, Carine Fortes Aragão, Valéria Cristina Soares Pinheiro

**Affiliations:** 1 Programa de Pós-Graduação em Biodiversidade e Biotecnologia—Rede de Biodiversidade e Biotecnologia da Amazônia Legal—BIONORTE, Universidade Estadual do Maranhão, São Luís 65081-400, Brazil; karen.brayner@discente.ufma.br (K.B.A.P.); rsobioq@hotmail.com (R.d.S.O.); 2 Departamento de Medicina, Universidade Estadual do Maranhão, Caxias 65600-000, Brazil; 3 Programa de Pós-Graduação em Biodiversidade, Ambiente e Saúde—PPGBAS, Universidade Estadual do Maranhão, Caxias 65600-000, Brazil; laercio1618@hotmail.com; 4 Instituto Evandro Chagas, Ananindeua 67030-000, Brazil; carinefaragao@gmail.com

**Keywords:** epidemiology, neglected diseases, public health surveillance

## Abstract

Background: Human visceral leishmaniasis remains a serious public health problem in Brazil, especially in the Northeast, where transmission persists in various vulnerable settings. Objective: This study aimed to assess temporal trends in the incidence of human visceral leishmaniasis in Maranhão, northeastern Brazil, with emphasis on social vulnerability and regional heterogeneity. Methods: We conducted an ecological, population-based study using confirmed cases of human visceral leishmaniasis reported in Maranhão from 2007 to 2024. Case data were obtained from the Brazilian Notifiable Diseases Information System, and population denominators were derived from national census counts and annual population estimates. Temporal trends in incidence rates were analyzed using joinpoint regression models, stratified by sex, age group, Social Vulnerability Index, and Health Regions. Results: A total of 7830 new cases of human visceral leishmaniasis were reported during the study period. Incidence showed a significant upward trend from 2007 to 2017, followed by a marked decline from 2018 to 2024. A heterogeneous pattern was observed in the average incidence rate of human visceral leishmaniasis across municipalities’ vulnerability categories, as measured by each subindex of the Social Vulnerability Index. Substantial regional heterogeneity was identified, with marked reductions in incidence in the Health Regions of Caxias, Timon, Barra do Corda, and Codó, whereas Santa Inês and Viana showed increasing trends. Conclusions: Despite the decline in incidence, human visceral leishmaniasis remains unevenly distributed throughout Maranhão. These findings underscore the need for geographically targeted interventions and the expansion of public health strategies aimed at preventing and controlling the disease.

## 1. Introduction

Human visceral leishmaniasis (HVL) is a neglected tropical zoonosis caused by protozoa of the genus Leishmania and transmitted by the bite of infected sand flies [1]. The disease presents a serious public health challenge worldwide, especially in tropical and subtropical regions, due to its high lethality when not diagnosed and treated at an early stage [2].

In the Americas, HVL is endemic in approximately 13 countries [3]. There were 74,440 cases of HVL reported in the Americas between 2001 and 2024, with over 95% of these cases occurring in Brazil. Brazil accounts for the vast majority of reported cases in the Americas, and the Northeast region remains the main endemic focus of the disease [4]. In this context, processes such as unplanned urbanization, land-use change, and environmental transformation have intensified over time, modifying vector habitats and patterns of human exposure. As a result, these factors have contributed to the emergence and persistence of HVL transmission in peri-urban and urban settings [5].

HVL remains a major public health challenge in Brazil, particularly in regions affected by poverty and inadequate living conditions [6,7,8]. Understanding the temporal dynamics of the disease is therefore essential to guide surveillance strategies and optimize control efforts. Two recent studies have analyzed HVL trends in Brazil, focusing on HVL–HIV/AIDS coinfection and incidence patterns by age and sex [9,10]. Although these investigations have advanced the understanding of HVL epidemiology, they have primarily addressed clinical and demographic aspects, without incorporating measures of social vulnerability. Consequently, important gaps remain in knowledge regarding long-term HVL incidence patterns in socially vulnerable populations. This study aims to address this gap by assessing temporal trends in HVL incidence in areas of high social vulnerability in northeastern Brazil.

## 2. Materials and Methods

### 2.1. Study Design and Area

This is an ecological time-series study based on secondary data on HVL in the state of Maranhão, Brazil, from 2007 to 2024. The units of observation consist of population groups or territorial areas that are systematically monitored over defined time periods [11].

**Figure 1:**
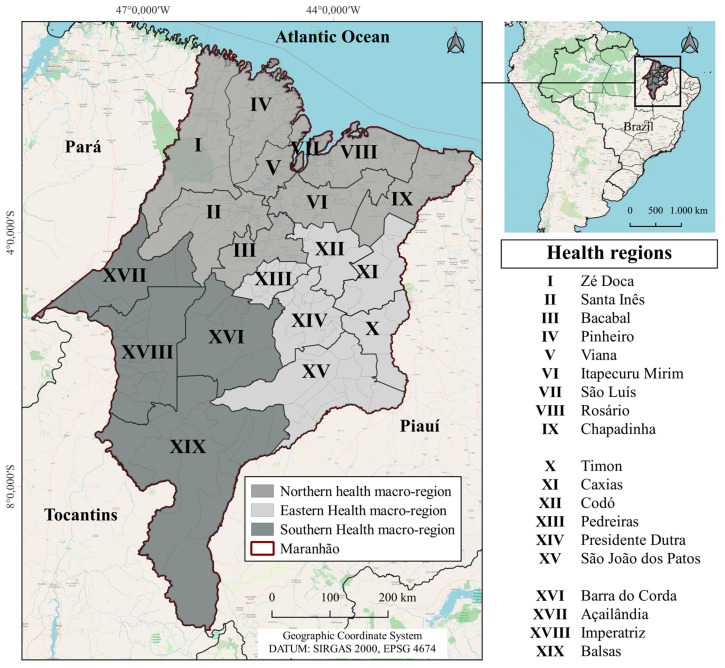
The state of Maranhão, Brazil.

The study area is the state of Maranhão, situated in northeastern Brazil. The state spans 329,651.478 km^2^ and, according to the 2022 national demographic census, is home to 6,776,699 inhabitants, corresponding to a population density of 20.56 inhabitants per km^2^. From an administrative standpoint, Maranhão is subdivided into 217 municipalities and, within the public health system, is organized into 19 Health Regions: Açailândia, Bacabal, Barra do Corda, Balsas, Caxias, Chapadinha, Codó, Imperatriz, Itapecuru-Mirim, Pedreiras, Pinheiro, Presidente Dutra, Rosário, Santa Inês, São João dos Patos, São Luís, Timon, Viana, and Zé Doca (Figure 1) [12,13].

### 2.2. Data Source

In Brazil, HVL is a notifiable disease, which ensures systematic and continuous surveillance of its occurrence throughout the national territory. Information on reported cases was obtained from the electronic platform of the Department of Information Technology of the Unified Health System (DATASUS) (http://www2.datasus.gov.br; accessed on 20 November 2025 at 10:21), specifically from the Notifiable Diseases Information System (SINAN), which consolidates official records of notifiable diseases and conditions in the country. The population denominators were obtained from official demographic data provided by *Instituto Brasileiro de Geografia e Estatística* (IBGE) (https://www.ibge.gov.br/estatisticas/sociais/populacao/25089-censo-1991-6.html?edicao=25091; accessed on 21 November 2025 at 16:43), including resident population counts based on the 2010 and 2022 censuses and the corresponding official annual population estimates for the other years of the study period. No interpolation or extrapolation procedures were performed by the authors. Social Vulnerability Index (SVI) data were obtained from the digital platform of the *Instituto de Pesquisa Econômica Aplicada* (IPEA) (https://ivs.ipea.gov.br/#/, accessed on 20 November 2025 at 13:20) [14], based on data from the 2010 IBGE Census.

### 2.3. Study Variables and Statistical Analysis

The HVL incidence rate was calculated as the ratio between the number of reported cases from 2007 to 2024 and the corresponding resident population for each year of occurrence. Rates were standardized and expressed as cases per 100,000 inhabitant-years. Municipalities were classified according to the Social Vulnerability Index (SVI) and its sub-indices (urban infrastructure, human capital, and income/work) into five categories: very low, low, medium, high, and very high vulnerability. Average incidence rates were calculated for each category, enabling comparison of epidemiological patterns according to different dimensions of social vulnerability.

The temporal trends in incidence rates were examined using regression models that allow the adjustment of segmented log-linear regression functions to the data, in which each segment represents a distinct trend interval, connected by inflection points called joinpoints. For the trend analysis, we sought to identify the regression model that best described the relationship between the independent variable (year) and the dependent variable (confirmed HVL cases). To define the final model, the software first performed a sequence of permutation tests based on the null hypothesis (H0) to determine the most appropriate number of inflection points in the time series. In addition, the Bayesian Information Criterion (BIC) was used to compare competing models. The model with the lowest BIC value was selected as the best-fitting model. This approach makes it possible to assess whether a segmented regression model, composed of multiple linear segments, provides a statistically better description of the temporal evolution of the disease than a simple linear model or models with fewer segments [15].

The magnitude and direction of the trends were expressed using the Annual Percentage Change (APC) and the Average Annual Percentage Change (AAPC), both accompanied by 95% confidence intervals (95% CIs). The APC estimates the average annual change in the rate in each segment and is considered statistically significant when the 95% CI does not include zero. In this sense, positive APC values with a 95% CI above zero indicate an upward trend, while negative values with a 95% CI below zero characterize a downward trend. When the 95% CI encompasses zero, the trend is interpreted as stable, suggesting an absence of statistically significant variation over the analyzed period.

The analyses were stratified by sex, age group, Health Regions, and categories of the SVI, including the overall SVI and its component dimensions of Urban Infrastructure, Income and Work, and Human Capital. SVI values range from 0 to 1, with higher values indicating greater social vulnerability. For analytical purposes, SVI scores were classified into five categories according to the official methodology of the Brazilian Atlas of Social Vulnerability: very low (SVI ≤ 0.200), low (0.201–0.300), medium (0.301–0.400), high (0.401–0.500), and very high (SVI > 0.500) [14]. All analyses adopted a significance level of 5%, and trend classification followed the standardized criteria of the Joinpoint Regression Program developed by the National Cancer Institute (Rockville, MD, USA), version 4.5.0.1.

### 2.4. Legal and Ethical Aspects of Research

This study was based on publicly available secondary data, with no individual identification of participants, and obtaining informed consent was not required. The study adhered to the ethical principles outlined in the Declaration of Helsinki and complied with Brazilian regulations for research involving human subjects, as established by Resolution No. 466 of 12 December 2012 and Resolution No. 510 of 7 April 2016, both issued by the Brazilian National Health Council [16,17].

## 3. Results

**Figure 2:**
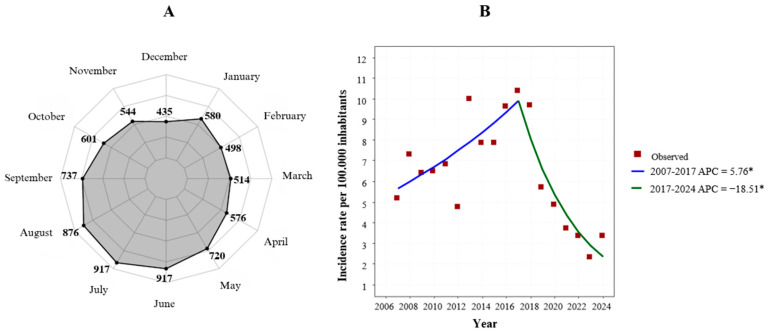
Monthly distribution of cases and incidence trends of human visceral leishmaniasis in Maranhão, Brazil, 2007–2024. Note: (**A**). Joinpoint regression analysis of incidence rates of human visceral leishmaniasis. (**B**). Monthly distribution of reported cases of human visceral leishmaniasis. * Indicates that the Annual Percent Change (APC) is significantly different from zero at the alpha = 0.05 level.

From January 2007 to December 2024, a total of 7830 new cases of HVL were confirmed. Incidence peaked in July (917 cases; 11.71%) and August (876 cases; 11.19%), whereas the lowest frequencies were observed in December (435 cases; 5.56%) and February (498 cases; 6.36%). Joinpoint regression analysis of incidence rates identified a significant increasing trend between 2007 and 2017, with an APC of 5.76% (95% CI 1.26 to 10.45; *p* = 0.0154), followed by a marked decline from 2018 to 2024 (APC −18.51%; 95% CI −25.49 to −10.87; *p* = 0.0002). Over the entire study period, incidence rates exhibited an overall decreasing trend, with an AAPC of −5.01% (95% CI −8.79 to −1.06; *p* = 0.0133) (Figure 2).

Joinpoint regression models stratified by sex showed a statistically significant initial increasing trend only among males (APC 6.77%; 95% CI 1.88 to 11.89), whereas the subsequent decline was significant for both sexes and was slightly more pronounced among females (APC −20.92%; 95% CI −28.23 to −12.87). When stratified by age group, the largest annual increase during the first period was observed among individuals aged 60 years or older (APC 18.12%; 95% CI 7.69 to 29.55). In contrast, the steepest decline during the second period occurred among children aged 1–4 years (APC −29.53%; 95% CI −42.84 to −13.13). All age groups exhibited statistically significant decreasing trends between 2018 and 2024, with annual reductions ranging from 7.69% in those aged 40–59 years to 29.53% among those aged 1–4 years (Table 1).

Over the entire period, the average incidence rates of HVL were higher in municipalities with low (8.18 per 100,000 inhabitants) and high (7.09 per 100,000 inhabitants) overall social vulnerability, respectively. Municipalities classified as having a medium overall SVI showed an upward trend from 2007 to 2016 (APC = 6.85; 95% CI: 0.64–13.44; *p* < 0.05). Municipalities classified as having a very high overall SVI showed an upward trend from 2007 to 2017 (APC = 8.68; 95% CI: 2.90–14.79; *p* < 0.05). In the IVS Urban Infrastructure subindex, the average incidence rate was higher in municipalities with medium vulnerability (10.05 per 100,000 inhabitants); an upward trend was observed from 2007 to 2017 in municipalities with high vulnerability (APC = 11.05; 95% CI: 5.57; 16.81; *p* < 0.05) and from 2007 to 2018 in municipalities with very high vulnerability (APC = 7.35; 95% CI: 3.03; 11.85; *p* < 0.05). In the IVS income and labor subindex, only municipalities with very high vulnerability showed an upward trend from 2007 to 2017 (APC = 7.97; 95% CI: 2.70; 13.50, *p* < 0.05). Regarding the human capital subindex, an upward trend was observed in municipalities with very high vulnerability from 2007 to 2017 (APC = 6.45; 95% CI: 1.32; 11.83, *p* < 0.05). Regarding the income/work and human capital domains, the average incidence rate was higher in municipalities with a high IVS (7.81 per 100,000 inhabitants and 8.53 per 100,000 inhabitants, respectively) (Table 2).

Regarding Health Regions, trend analyses indicated that four regions experienced significant reductions in incidence, including Caxias (AAPC −10.57; 95% CI: −15.38 to −5.49), Timon (AAPC −9.02; 95% CI: −15.23 to −2.37), Barra do Corda (AAPC −7.35; 95% CI: −13.90 to −0.30), and Codó (AAPC −7.19; 95% CI: −10.53 to −3.72). Santa Inês (AAPC 23.39; 95% CI: 8.63 to 40.19) and Viana (AAPC 16.43 *; CI 10.44 to 22.74) showed an increasing trend in incidence (Table 3).

**Figure 3:**
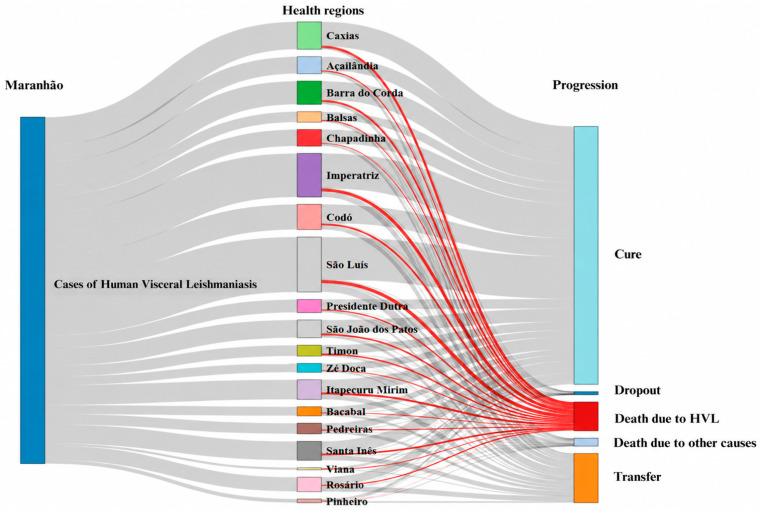
Case outcomes of human visceral leishmaniasis (HVL) by Health Region in Maranhão, Brazil, 2007–2024. Sankey diagram illustrating the distribution of case evolution. Color codes distinguish between different types of case flows; the gray bars indicate the overall case flow and their respective outcomes, with a width proportional to the number of cases. The red bars specifically highlight the case flows that resulted in death from human visceral leishmaniasis.

Among reported HVL cases, 62.20% (*n* = 4870) progressed to cure, whereas 8.79% (*n* = 688) resulted in death, of which 6.95% (*n* = 544) were attributable to the disease. The São Luís Health Region accounted for the largest number of cases (*n* = 1065; 13.60%), followed by Imperatriz (*n* = 923; 11.79%) and Caxias (*n* = 742; 9.48%). The highest cure proportion was observed in the Caxias Health Region (*n* = 453; 86.78%). Cure rates above 80% were also recorded in Chapadinha (80.84%) and Barra do Corda (80.69%). In contrast, lower cure proportions were observed in Bacabal (59.65%), Pinheiro (55.71%), and Rosário (54.65%). Patient transfers were more frequent in the Bacabal (29.24%), Pinheiro (35.71%), and Rosário (36.06%) Health Regions (Figure 3).

## 4. Discussion

This study provides a comprehensive analysis of temporal trends, sociodemographic patterns, and regional heterogeneity of HVL over an 18-year period in Maranhão, one of the most socioeconomically vulnerable states in Brazil. By applying Joinpoint regression analyses stratified by sex, age group, social vulnerability, and Health Regions, our findings provide a detailed characterization of the evolving epidemiological dynamics of HVL in a historically endemic setting.

The incidence of HVL exhibited a well-defined seasonal pattern, with higher case concentrations in July and August. This pattern is likely associated with increased sand fly vector abundance and intensified transmission during dry periods following seasonal rainfall fluctuations in northeastern Brazil [18,19]. In Maranhão, this dynamic may be further amplified by the state’s location within a broad ecotonal zone, encompassing the Amazon Forest to the west, the Cerrado to the south and east, and transitional formations between the Caatinga and coastal ecosystems, including sandbanks, mangroves, and coastal dunes [20]. This diversity of vegetation types creates heterogeneous microenvironments that favor the persistence of sand fly populations and enhance connectivity between sylvatic and human reservoirs, thereby increasing the potential for disease transmission [21].

Social vulnerability, in the context of neglected tropical diseases, should not be understood as a single, homogeneous attribute, but rather as a set of interrelated dimensions, such as poverty, precarious housing, inadequate sanitation and urban services, low educational attainment, and barriers to accessing health services, among others, which can modify, to varying degrees, exposure, susceptibility, timely diagnosis, and the outcomes of these conditions [22,23]. In the case of HVL, the transmission and distribution of the disease tend to be concentrated in socially more vulnerable areas, especially where structural deficiencies and weaknesses in urban organization are concentrated. Similar patterns have also been described for other diseases, such as tuberculosis and COVID-19, although the magnitude and direction of these effects vary according to transmission dynamics, the natural history of the disease, and the response capacity of health services [24,25,26].

In Maranhão, HVL was first described on the island of São Luís in 1980 and was historically associated with rural, forested, and subsistence farming areas [27]. Since then, the disease has undergone a progressive process of urbanization across different regions of the state. This process may be further intensified by recent land-use and land-occupation changes, including the expansion of the agricultural frontier and the growth of ecotourism, particularly in the northern and southern regions, which increase population mobility and transient exposure to endemic areas, potentially influencing local transmission dynamics [28,29].

Trend analyses revealed substantial heterogeneity across Health Regions. Although most regions exhibited declining incidence trends, four regions, namely, Caxias, Timon, Barra do Corda, and Codó, showed more pronounced reductions. These patterns may reflect differences in the effectiveness of local control measures, epidemiological surveillance capacity, and access to healthcare services, as well as potential limitations in case detection and reporting. Evidence from other Brazilian states, including Piauí and Mato Grosso, suggests that the interaction between poverty, peripheral urbanization, and vector adaptation can sustain transmission and contribute to the persistence of endemic areas, even in contexts where incidence appears to be declining [30,31].

Since 2012, after the establishment of the National Leishmaniasis Control Week by Federal Law No. 12,604, Maranhão State has strengthened control campaigns in all 217 municipalities, particularly in priority areas and with a greater focus on vector control, which may have contributed to the subsequent decline in VL occurrence. These actions included expanded diagnosis of human and canine cases, distribution of insecticide-impregnated dog collars in high-incidence municipalities, training of health professionals in clinical management, enhanced monitoring of cases and deaths, and reinforced distribution of medicines and rapid diagnostic tests for both human and canine surveillance. Furthermore, the Action Plan for the Prevention and Control of Deforestation and Wildfires in the Cerrado biome of Maranhão may also have affected the eco-epidemiological context of transmission [32,33,34].

This study has limitations inherent to the use of secondary data from the SINAN, including underreporting, incomplete or duplicate records, and delays in case notification. The SVI was treated as a fixed contextual measure rather than a time-varying variable, as no comparable annual SVI estimates were available for the entire 2007–2024 period. We acknowledge that this approach may not fully capture temporal changes in municipal social vulnerability. Nevertheless, SINAN represents the primary national surveillance system for notifiable diseases and enables comprehensive population-based analyses over extended time periods and large geographic areas, providing valuable insights into temporal trends of HVL in Brazil [30].

These results reinforce the need for integrated strategies to control HVL, with an emphasis on strengthening local and intermunicipal management, epidemiological surveillance, and coordination across different levels of healthcare. In this context, sustainable control of the disease is closely aligned with the Sustainable Development Goals (SDGs) of the 2030 Agenda, particularly SDG 3 (Goal 3: Ensure healthy lives and promote well-being for all at all ages), SDG 1 (End poverty in all its forms everywhere), SDG 10 (Reduce inequality within and among countries), SDG 11 (Make cities inclusive, safe, resilient and sustainable), and SDG 13 (Take urgent action to combat climate change and its impacts) [35,36,37].

Prioritizing localized actions targeting Health Regions and socially vulnerable populations is essential to address the observed inequalities. In this context, primary healthcare represents the main entry point to the Unified Health System and should be strengthened as the foundation for prevention, early case detection, clinical follow-up, and care coordination [38,39]. Effective integration between primary healthcare, specialized services, and epidemiological surveillance is necessary to ensure coordinated care pathways, reduce morbidity and mortality, and support sustainable control of HVL at the state level, while also contributing to progress toward the Sustainable Development Goals of the 2030 Agenda [40,41].

## 5. Conclusions

In conclusion, this eighteen-year analysis of HVL in Maranhão identified a biphasic temporal pattern, with a significant increase in incidence from 2007 to 2017 followed by a marked decline from 2018 to 2024, as well as pronounced seasonal peaks in July and August. Substantial heterogeneity was observed across Health Regions, with pronounced reductions in incidence in Caxias, Timon, Barra do Corda, and Codó, contrasted with an increasing trend in Santa Inês. Together, these findings indicate that effective disease control in the state requires territorially targeted interventions, strengthened epidemiological surveillance, and improved integration across different levels of healthcare.

## Figures and Tables

**Table 1:** Trend analysis of human visceral leishmaniasis incidence (per 100,000 population) according to sociodemographic characteristics in Maranhão, Brazil, 2007–2024.

Variables		1st Trend			2nd Trend	
	Period	APC ^a^	CI 95% ^b^	Period	APC ^a^	CI 95% ^b^
Sex						
General	2007–2017	5.76 *	1.26; 10.45	2017–2024	−18.51 *	−25.49; −10.87
Male	2007–2017	6.77 *	1.88; 11.89	2017–2024	−17.3429 *	−24.58; −9.42
Female	2007–2017	4.07	−0.24; 8.57	2017–2024	−20.9219 *	−28.23; −12.87
Age groups (years)						
≤1 year	2007–2017	8.73 *	2.01; 15.88	2017–2024	−24.93 *	−35.70; −12.33
1 to 4	2007–2018	4.33	−0.44; 9.32	2018–2024	−29.53 *	−42.84; −13.13
5 to 9	2007–2017	2.78	−1.52; 7.27	2017–2024	−20.61 *	−29.11;−11.09
10 to 19	2007–2017	7.21 *	2.58; 12.06	2017–2024	−19.94 *	−27.59; −1.47
20 to 39	2007–2017	8.15 *	3.53; 12.97	2017–2024	−15.53 *	−22.34; −8.13
40 to 59	2007–2016	13.01 *	7.94; 18.31	2016–2024	−7.69 *	−11.56; −3.66
60 or more	2007–2016	18.12 *	7.69; 29.55	2016–2024	−11.34 *	−18.17; −3.94

Note: ^a^ APC: Annual Percent Change. ^b^ CI: Confidence interval. * *p*-value < 0.05.

**Table 2:** Incidence rates of human visceral leishmaniasis (per 100,000 population) according to Social Vulnerability Index classification, Maranhão, Brazil, 2007–2024.

SVI General	Number of Municipalities	Average Incidence	Period	APC ^a^	CI 95% ^b^	Trend
Very low	-	-	-	-	-	-
Low	1	8.18	2007–2024	−9.00 *	−12.02; −5.89	Decreasing
Medium	9	5.60	2007–2016	6.85 *	0.64; 13.44	Increasing
			2016–2024	−16.79 *	−23.73; −9.23	Decreasing
High	32	7.09	2007–2017	−0.02	−3.42; 3.48	Stationary
			2017–2024	−18.52 *	−25.12; −11.35	Decreasing
Very high	167	6.43	2007–2017	8.68 *	2.90; 14.79	Increasing
			2017–2024	−18.31 *	−26.15; −9.65	Decreasing
SVI Urban Infrastructure	Number of municipalities	Average incidence	Period	APC ^a^	CI 95% ^b^	Trend
Very low	6	8.07	2007–2024	−5.42 *	−8.09; −2.69	Decreasing
Low	17	8.69	2007–2018	−0.39	−4.75; 4.16	Stationary
			2018–2024	−23.21 *	−35.37; −8.75	Decreasing
Medium	16	10.05	2007–2014	1.14	−6.14; 8.99	Stationary
			2014–2024	−18.41 *	−24.48; −11.87	Decreasing
High	37	5.06	2007–2017	11.05 *	5.57; 16.81	Increasing
			2017–2024	−21.34 *	−28.85; −13.02	Decreasing
Very high	133	5.89	2007–2018	7.35 *	3.03; 11.85	Increasing
			2018–2024	−19.74 *	−28.73; −9.62	Decreasing
SVI Income and Work	Number of municipalities	Average incidence	Period	APC ^a^	CI 95% ^b^	Trend
Very low	-	-	-	-	-	-
Low	-	-	-	-	-	-
Medium	7	5.30	2007–2017	3.03	−1.72; 8.00	Stationary
			2017–2024	−18.94 *	−27.35; −9.57	Decreasing
High	31	7.81	2007–2016	0.92	−4.65; 6.83	Stationary
			2016–2024	−13.17 *	−19.97; −5.78	Decreasing
Very high	171	6.57	2007–2017	7.97 *	2.70; 13.50	Increasing
			2017–2024	−18.87 *	−26.29; −10.69	Decreasing
SVI Human Capital	Number of municipalities	Average incidence	Period	APC ^a^	CI 95% ^b^	Trend
Very low	-	-	-	-	-	-
Low	1	3.45	2007–2009	−30.59	−66.86; 45.35	Stationary
			2009–2016	24.52 *	11.05; 39.63	Increasing
			2016–2024	−18.72 *	−24.76; −12.19	Decreasing
Medium	3	7.33	2007–2024	−6.23 *	−9.19; −3.16	Decreasing
High	19	8.53	2007–2017	1.55	−3.92; 7.34	Stationary
			2017–2024	−18.32 *	−28.03; −7.29	Decreasing
Very high	186	6.64	2007–2017	6.45 *	1.32; 11.83	Increasing
			2017–2024	−18.49 *	−26.08; −10.13	Decreasing

Note: ^a^ APC: Annual Percent Change. ^b^ CI: Confidence interval. * *p*-value < 0.05.

**Table 3:** Trend analysis of human visceral leishmaniasis according to Health Regions in Maranhão, Brazil, 2007–2024.

Health Regions	Period	Trend		Overall Period	
		APC ^a^	CI _95%_ ^c^	AAPC ^b^	CI _95%_ ^c^
Açailândia	2007–2018	9.55 *	1.95; 17.69	−1.58	−8.64; 6.01
	2018–2024	−19.14 *	−33.22; −2.07		
Bacabal	2007–2024	−2.82	−8.81; 3.57	-	-
Balsas	2007–2017	14.56 *	6.56; 23.17	−6.16	−12.43; 0.56
	2017–2024	−29.44 *	−39.47; −17.73		
Barra do Corda	2007–2017	17.73 *	8.83; 27.36	−7.35 *	−13.90; −0.30
	2017–2024	−34.21 *	−43.99; −22.71		
Caxias	2007–2014	4.47	−3.56; 13.17	−10.57 *	−15.38; −5.49
	2014–2024	−19.79 *	−26.48; −12.49		
Chapadinha	2007–2018	11.02 *	3.49; 19.11	−1.35	−8.58; 6.47
	2018–2024	−20.54 *	−34.96; −2.95		
Codó	2007–2014	5.87	−0.74; 12.90	−7.19 *	−10.53; −3.72
	2014–2024	−15.35 *	−19.63; −10.85		
Imperatriz	2007–2024	−5.69 *	−8.69; −2.59	-	-
Itapecuru Mirim	2007–2024	−5.03	−9.88; 0.08	-	-
Pedreiras	2007–2024	−5.76 *	−9.48; −1.89	-	-
Pinheiro	2007–2024	0.75	−4.27; 6.03	-	-
Presidente Dutra	2007–2017	7.58	−1.65; 17.68	−4.61	−12.33; 3.79
	2017–2024	−19.66 *	−33.29; −3.23		
Rosário	2007–2013	46.36 *	15.27; 85.82	7.19	−1.53; 16.68
	2013–2024	−9.56 *	−15.05; −3.71		
Santa Inês	2007–2013	115.29 *	46.79; 215.74	23.39 *	8.63; 40.19
	2013–2024	−8.92 *	−14.19; −3.31		
São João dos Patos	2007–2017	8.14	−2.68; 20.16	−6.09	−15.09; 3.88
	2017–2024	−23.23 *	−38.63; −3.96		
São Luís	2007–2010	−20.07	−48.27; 23.51	−5.77	−13.75; 2.95
	2010–2016	24.44 *	7.05; 44.67		
	2016–2024	−18.65 *	−24.87; −11.91		
Timon	2007–2013	16.68 *	0.36; 35.64	−9.03 *	−15.23; −2.37
	2013–2024	−20.57 *	−27.25; −13.29		
Viana	2007–2024	16.43 *	10.44; 22.74	-	-
Zé Doca	2007–2018	30.09 *	10.79; 52.76	9.11	−3.33; 23.16
	2018–2024	−20.96	−37.62; 0.17		

Note: ^a^ APC: Annual Percent Charge. ^b^ AAPC: Average annual percentage variation ^c^ CI: Confidence interval. * *p*-value < 0.05.

## Data Availability

No new data were generated for this study. All data underlying the findings are publicly available from open-access repositories and published sources cited in the manuscript.

## Abbreviations

The following abbreviations are used in this manuscript:

HVL	Human visceral leishmaniasis
HIV	Human immunodeficiency virus
AIDS	Acquired immunodeficiency syndrome
DATASUS	Department of Information Technology of the Unified Health System
SINAN	Notifiable Diseases Information System
SVI	Social Vulnerability Index
APC	Annual Percentage Change
AAPC	Average Annual Percentage Change
CI	Confidence intervals
SDGs	Sustainable Development Goals
